# *Lactobacillus paracasei* CCFM1223 Protects against Lipopolysaccharide-Induced Acute Liver Injury in Mice by Regulating the “Gut–Liver” Axis

**DOI:** 10.3390/microorganisms10071321

**Published:** 2022-06-30

**Authors:** Weiling Guo, Bingyong Mao, Xin Tang, Qiuxiang Zhang, Jianxin Zhao, Shumao Cui, Hao Zhang

**Affiliations:** 1 State Key Laboratory of Food Science and Technology, Jiangnan University, Wuxi 214122, China; 7200112062@stu.jiangnan.edu.cn (W.G.); maobingyong@jiangnan.edu.cn (B.M.); xintang@jiangnan.edu.cn (X.T.); zhangqx@jiangnan.edu.cn (Q.Z.); zhaojianxin@jiangnan.edu.cn (J.Z.); zhanghao61@jiangnan.edu.cn (H.Z.); 2 School of Food Science and Technology, Jiangnan University, Wuxi 214122, China; 3 National Engineering Research Center for Functional Food, Jiangnan University, Wuxi 214122, China

**Keywords:** *Lactobacillus paracasei* CCFM1223, acute liver injury, gut–liver axis, intestinal microbiota, mRNA expression

## Abstract

Background: *Lactobacillus paracasei* CCFM1223, a probiotic previously isolated from the healthy people’s intestine, exerts the beneficial influence of preventing the development of inflammation. Methods: The aim of this research was to explore the beneficial effects of *L. paracasei* CCFM1223 to prevent lipopolysaccharide (LPS)-induced acute liver injury (ALI) and elaborate on its hepatoprotective mechanisms. Results: *L. paracasei* CCFM1223 pretreatment remarkably decreased the activities of serum aspartate aminotransferase (AST) and alanine aminotransferase (ALT) in mice with LPS treatment and remarkably recovered LPS-induced the changes in inflammatory cytokines (tumor necrosis factor-α (TNF-α), transforming growth factor-β (TGF-β), interleukin (IL)-1β, IL-6, IL-17, IL-10, and LPS) and antioxidative enzymes activities (total antioxidant capacity (T-AOC), superoxide dismutase (SOD), glutathione peroxidase (GSH-Px), and catalase (CAT)). Metagenomic analysis showed that *L. paracasei* CCFM1223 pretreatment remarkably increased the relative abundance of *Catabacter* compared with the LPS group but remarkably reduced the relative abundance of [*Eubacterium*] *xylanophilum* *group*, *ASF356*, *Lachnospiraceae* *NK4A136* *group*, and *Lachnoclostridium*, which is closely associated with the inflammation cytokines and antioxidative enzymes. Furthermore, *L. paracasei* CCFM1223 pretreatment remarkably increased the colonic, serum, and hepatic IL-22 levels in ALI mice. In addition, *L. paracasei* CCFM1223 pretreatment remarkably down-regulated the hepatic Tlr4 and Nf-kβ transcriptions and significantly up-regulated the hepatic Tlr9, Tak1, Iκ-Bα, and Nrf2 transcriptions in ALI mice. Conclusions: *L. paracasei* CCFM1223 has a hepatoprotective function in ameliorating LPS-induced ALI by regulating the “gut–liver” axis.

## 1. Introduction

The liver serves as the most important organ responsible for energy and drug metabolism, thereby leading to it vulnerable to damage. The case of liver-associated diseases is quickly rising, and it has been reported to be the cause of 2 million people dying worldwide every year [1]. Among those, acute liver injury (ALI) is a complicated and health-threatening disease that results from multiple environmental factors, such as alcohol consumption, abuse of drugs, and endotoxemia. Despite the advances in medical conditions, treatment for endotoxemia-induced ALI maintains a challenge in clinical practice result from limited therapeutics. Lipopolysaccharide (LPS) acts as one of the common endotoxemia that major stem from Gram-negative bacteria. LPS promotes the secretion of inflammatory cytokines (tumor necrosis factor-α (TNF-α), interleukin (IL)-1β, and IL-6) and oxidative stress [malondialdehyde (MDA)] by activating toll-like receptor (Tlr) 4 and suppressing nuclear factor-E2-related factor 2 (Nrf2), and then destroying the intestinal barrier structure and accelerating the development of diseases-related liver [2]. Therefore, LPS is widely used in the establishment of the ALI model because its symptoms are similar to the clinical symptoms of ALI patients. The pathogenesis of ALI involves multiple complicated developments, such as inflammation, oxidative stress, and apoptosis [3]. Among these, it is well accepted that excessive inflammatory responses and oxidative stress are the most important factor in the occurrence and process of ALI. Patients with ALI exhibited higher levels of proinflammatory cytokines (TNF-α, IL-1β, and IL-6), lower levels of anti-inflammatory cytokines (IL-10), and lower activities of antioxidative enzymes (total antioxidant capacity (T-AOC), superoxide dismutase (SOD), glutathione peroxidase (GSH-Px), and catalase (CAT)) [4]. Therefore, inhibition of inflammatory responses and oxidative stress is considered an effective approach for the treatment of ALI.

*Lactobacillus* serves as one of the common probiotics that have previously attracted more and more attention because it provides health benefits. *Lactobacillus* is a facultative anaerobic bacteria that wide-range distributed over human intestines and traditional fermented food. Two strains of *Lactobacillus* were isolated from feces sampled from breastfed babies by Ding et al. [5]. In addition, some strains of *Lactobacillus* were isolated from the traditional brewing process of Hongqu rice wine [6]. Recently years, *Lactobacillus* has been gradually confirmed to avoid the intestinal flora disorder induced by endotoxemia, prevent the excessive inflammatory responses, and maintain the antioxidative enzyme activities [7]. Zhou and colleagues found that *L. fermentum* CQPC04 intervention effectively suppresses the secretion of TNF-α, IFN-γ, IL-1β, IL-6, and IL-12 and attenuates the enzyme activities of total superoxide dismutase (T-SOD), CAT induced by dextran sulfate sodium [8]. *L. plantarum* C88 has been reported to alleviate liver injury by reducing the activities of plasma biochemical markers (ALT and AST) and the secretion of TNF-α, IL-6, and IL-12 [9]. At present, the research on the anti-inflammatory and antioxidative efficacy of *Lactobacillus* in ALI mice mainly focuses on the Nf-kβ and Nrf2 pathways [10,11]. However, how the intestinal *Lactobacillus* regulates the hepatic Nf-kβ and Nrf2 pathways is unclear. Recently, the “gut–liver” axis has attracted wide attention in the world. A previous report exhibited that commensal *Lactobacillus* promotes the secretion of IL-22 via stimulating gut innate lymphoid cells and elevating the serum IL-22 concentrations [12]. Gut-derived IL-22 elevates the intestinal barrier integrity and accelerates the accumulation and proliferation of regulatory classical dendritic cells (cDCs) in the liver [13]. The accumulation of regulatory cDCs is beneficial for inhibiting the Nf-kβ pathway and activating the Nrf2 pathway and then improving the inflammation responses and oxidative stress.

*L. paracasei* is reported to have health-promoting attributes that have generated global interest in the food and pharmaceutical industries. *L. paracasei* CCFM1223 was isolated and purified from human intestines in this study. Nevertheless, it remains unknown whether *L. paracasei* CCFM1223 has health-promoting effects. Consequently, the aim of this experiment was to analyze the hepatoprotective efficacy of *L. paracasei* CCFM1223 in ALI mice from the perspective of inflammation responses and oxidative stress. Moreover, whether *L. paracasei* CCFM1223 intervention can shift the intestinal microbiota structure and stimulate the production of IL-22 was also explored using high-throughput sequencing and an ELISA kit. The results of this research supply a scientific basis for practical applications, purposing to relieve ALI and developing dietary supplements.

## 2. Materials and Methods

### 2.1. Materials

LPS (*Escherichia coli* 055:B5) were obtained from Aladdin (Beijing, China). De Man, Rogosa, and Sharpe (MRS) was from Solarbio Ltd. (Solarbio, Beijing, China). Total cholesterol (TC), triglyceride (TG), low-density lipoprotein cholesterol (LDL-C), high-density lipoprotein cholesterol (HDL-C), ALT, AST, MDA, T-AOC, SOD, GSH-Px, CAT, TNF-α, transforming growth factor (TGF)-β, IL-1β, IL-6, IL-17, IL-10, IL-22, and LPS kits were obtained from MY BioSource Co., Ltd. (San Diego, CA, USA).

### 2.2. Bacterial Strain Cultures

*L. paracasei* CCFM1223 was transferred to MRS agar plates and grown in the incubator at 37 °C for 24 h. Then, one *L. paracasei* CCFM1223 colony was inoculated with MRS broth and placed at 37 °C. After the 18 h of inoculation, *L. paracasei* CCFM1223 were transferred (1:25) to the fresh culture media. After the 18 h of inoculation, the sediments were obtained by centrifugation (8000 rpm, 10 min, 4 °C), followed by washing three times with PBS. The sediments were resuspended using 0.85% saline and the final concentration of 5.0 × 10^9^ CFU/mL.

### 2.3. Animal Experiment

Twenty-four 7-week-old male C57BL/6J mice were obtained from Animal Research Center (Hangzhou, China) and raised in a controlled room with about 22.0 °C and unrestricted access to standard diet and water. After acclimating for one week, all mice were arbitrarily distributed to three groups, namely the control (NC), model (LPS), and experiment (LPS+Lp) groups (Figure S1). The mice in the NC and LPS groups were given 0.2 mL saline every day, whereas the mice in the LPS+Lp group were given with equal volume of *L. paracasei* CCFM1223 solution (1.0 × 10^9^ CFU) every day. After 14 days of the experiment, the mice in the LPS and LPS+Lp groups were treated with an intraperitoneal injection of 0.2 mL LPS (5 mg/kg) after fasting for 6 h, whereas mice in the NC group were treated by intraperitoneal injection of 0.2 mL saline. All mice were sacrificed 4 h after LPS treatment. The experiment was approved by the Jiangnan University Animal Ethics Committee (JN.No20211130S0360525[491]).

### 2.4. Blood, Liver, and Colon Analysis

The blood was obtained through the heart and placed at ordinary temperature for 1.0~1.5 h. After 15 mins of centrifugation (3500 rpm, 25 °C), all serum was individually collected and stored at −72 °C in a refrigerator. The serum TC, TG, LDL-C, HDL-C, ALT, AST, and IL-22 levels were measured using a commercial kit (MY BioSource, CA, USA).

Liver tissues were harvested and homogenized with 0.85% saline or RIPA Lysis Buffer at a ratio of 1:9. After 10 mins of centrifugation (13,000 rpm, 4 °C), the supernatant was collected and analyzed. The concentrations of hepatic MDA, T-AOC, SOD, GSH-Px, CAT, TNF-α, TGF-β, IL-1β, IL-6, IL-17, IL-10, IL-22, and LPS were measured using a commercial kit (MY BioSource, CA, USA).

Colon tissues were harvested and homogenized with RIPA Lysis Buffer at a ratio of 1:9. After 10 mins of centrifugation (13,000 rpm, 4 °C), the supernatant was collected and analyzed. The concentrations of colonic IL-22 were measured using a commercial kit (MY BioSource, CA, USA).

### 2.5. Hematoxylin and Eosin (H&E) Staining

Partial liver tissues were collected and fixed with a 4% formaldehyde solution for 1 day, washed with water for 2 h, and then embedded in paraffin. All samples were cut into 2~4 μm thick uniform slices and hematoxylin-eosin staining (H&E). Finally, the sections were observed and photographed using 3Dhistech Pannoramic Scan (Budapest, Hungary).

### 2.6. Immunohistochemistry

Immunohistochemical analysis was carried out as described in the previous report [14]. Briefly, partial liver tissues were harvested and incubated with a primary antibody to p65, Iκ-Bα, and Nrf2 for 60 min and then incubated with a secondary antibody and streptavidin-biotin peroxidase for 120 min. Ultimately, the brown color was carried out under 3,3′-diaminobenzidine tetrahydrochloride, following the manufacturer’s protocol. Counterstaining was carried out by Mayer’s hematoxylin (Abcam). Sections were observed using a panoramic scanner (Pannoramic MIDI, 3D HISTECH, Hungary).

### 2.7. Cecal Short-Chain Fatty Acids Analysis

The cecal contents were extracted and placed in a −72 °C refrigerator. The water of cecal contents was removed using freeze-drying. Then, 500 μL of NaCl solution and 100 mg cecal contents were mixed and placed at ordinary temperature for 60 min, followed by 20 mL H_2_SO_4_ (10%, *v/v*) and 800 μL C_2_H_5_OC_2_H_5_ added to the sample and vibrated. After centrifugation (13,000 rpm, 20 min, 4 °C), the supernatant was collected and placed in new centrifuge tubes (containing 0.25 g Na_2_SO_4_). After centrifugation (13,000 rpm, 20 min, 4 °C), the supernatant was collected and analyzed using gas chromatography (Thermo Fisher Scientific, Carlsbad, CA, USA).

### 2.8. Real-Time Quantitative PCR Analysis

Total RNA of the liver was extracted using the Trizol reagen (Sangon Biotech, Shanghai, China). The concentrations and quality of RNA were detected by a nanodrop 2000 spectrophotometer (Thermo Fisher Scientific, CA, USA). The RNA was reverse-transcribed into cDNA by a commercial kit (Vazyme, Nanjing, China). Subsequently, the cDNA was amplified by a real-time PCR instrument (Bio-Rad, Hercules, CA, USA). The amplification procedure: initial activation at 95 °C for 30 s, denaturation at 95 °C for 5 s, annealing at 58 °C for 30 s, and extension at 72 °C for 35 s, and the reaction was implemented for 40 cycles. The expressions of mRNA were calculated according to the expression of β-actin and the 2^−ΔΔCt^ method, and the primer sequences in the present study are shown in Table S1.

### 2.9. Intestinal Microbiota Analysis

Bacterial DNA from the feces sample was obtained by a commercial kit (Majorbio, Shanghai, China) according to the manufacturer’s instructions. Total DNA was amplified using broad-range bacterial primers (341F and 805R) and then purified using agarose gel electrophoresis (1.5% *w/v* agarose) and Agencourt AMPure XP Kit (Beckman Coulter, USA). The concentrations of bacterial DNA were measured using a nanodrop 2000 spectrophotometer (Thermo Fisher Scientific, CA, USA). All samples were mixed with equal concentrations, and the mixture was quantified using a nanodrop 2000 spectrophotometer and sequenced on a MiSeq platform (Illumina, CA, USA). Raw data were filtered and merged by Usearch (11.0), and the sequences with ≥97% similarity were assigned to the same operational taxonomic unit (OTU). Multiple diversity index analysis based on OUT was analyzed using Xshell 7.0, and principal coordinate analysis of intestinal flora was carried out using R software (4.1.2). The differences in intestinal microbiota between two groups using STAMP (version 2.1.3). The associations between the biochemical parameters and the intestinal microbiota were computed and visualized using R software (4.1.2) and Cytoscape (3.9.0).

### 2.10. Statistical Analysis

All data are presented as mean ± SD using GraphPad Prism (Ver. 7.0). The one-way ANOVA was firstly used, followed by Tuckey’s multiple-comparison test. Values with different letters are significantly different (*p <* 0.05).

## 3. Results

### 3.1. Effect of L. paracasei CCFM1223 on the Body Weight, Serum Biochemical Index in ALI Mice

**Figure 1:**
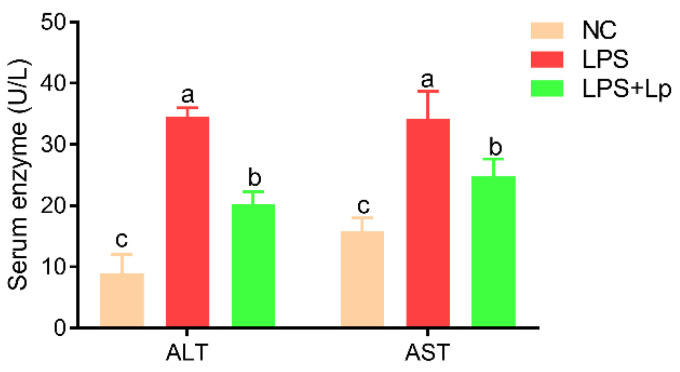
Effect of *L. paracasei* CCFM1223 pretreatment on serum ALT and AST levels in LPS-treated mice (n = 8). Values with different letters are significantly different (*p* < 0.05).

During the whole process of the experiment, the body weight of mice did not differ remarkably among the three groups (*p* > 0.05) (Figure S2). In addition, there were no remarkable differences in the serum TG, and HDL-C levels among the three groups (*p* > 0.05) (Figure S3). However, *L. paracasei* CCFM1223 pretreatment remarkably reduced the serum TC and LDL-C levels compared with that in the NC and LPS groups (*p* < 0.05). The serum ALT and AST activities of mice with LPS treatment were obviously higher than that of the NC group (*p* < 0.05) (Figure 1), suggesting the liver injury was successfully established. Interestingly, this trend was remarkably reversed after *L. paracasei* CCFM1223 pretreatment for 14 days (*p* < 0.05). These data indicated that *L. paracasei* CCFM1223 intervention improves the host energy metabolism and against ALI caused by LPS to a certain extent.

### 3.2. L. paracasei CCFM1223 Pretreatment Stimulated the IL-22 Production in ALI Mice

**Figure 2:**
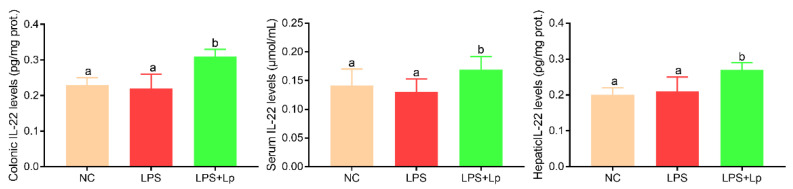
Effect of *L. paracasei* CCFM1223 pretreatment on the colonic, serum, and hepatic IL-22 levels in LPS-treated mice (n = 8). Values with different letters are significantly different (*p* < 0.05).

Gut-derived IL-22 improves the symptoms of ALI by the “gut–liver” axis; thus, the colonic, serum, and hepatic IL-22 levels were detected (Figure 2). There was no obvious difference in the colonic, serum, and hepatic IL-22 levels between the NC and LPS groups (*p* > 0.05). However, the colonic, serum, and hepatic IL-22 levels were obviously elevated in the LPS+Lp group compared with that in the NC and LPS groups (*p* < 0.05), suggesting that *L. paracasei* CCFM1223 treatment is beneficial for stimulating the IL-22 production in the colon.

### 3.3. L. paracasei CCFM1223 Pretreatment Inhibited the Inflammation and Oxidation Stress in ALI Mice

To further explore the anti-inflammatory and antioxidative effects of *L. paracasei* CCFM1223 pretreatment on ALI mice, the inflammatory cytokines production and antioxidative enzyme activities were emphatically analyzed (Table 1). Compared with the NC group, intraperitoneal injection of LPS obviously elevated the hepatic TNF-α, IL-1β, IL-17, and LPS levels (*p* < 0.05) and obviously reduced the hepatic IL-10 and TGF-β levels (*p* < 0.05), implying the ALI model were successfully established. There was no significant change in the hepatic IL-6 levels between the NC and LPS groups (*p* > 0.05). Interestingly, *L. paracasei* CCFM1223 pretreatment obviously suppressed the LPS-stimulated production of hepatic TNF-α, IL-1β, IL-6, IL-17, and LPS (*p* < 0.05) and accelerated the production of hepatic IL-10 and TGF-β (*p* < 0.05). Moreover, oxidative stress is frequently used in assessing liver function injury. The hepatic MDA levels (1.82 ± 0.65 nmol/mg prot.) of mice in the LPS group were remarkably higher than that (1.03 ± 0.33 nmol/mg prot.) of the NC group (*p* < 0.05). Nevertheless, the abnormal level of hepatic MDA (0.79 ± 0.30 nmol/mg prot.) was obviously ameliorated by pretreatment with *L. paracasei* CCFM1223 (*p* < 0.05). Intraperitoneal injection of LPS obviously suppressed the hepatic SOD, CAT, and T-AOC activities compared with that in the NC group (*p* < 0.05), whereas the hepatic GSH-Px activity was slightly reduced in the LPS group (*p* > 0.05). Unexpectedly, *L. paracasei* CCFM1223 pretreatment obviously protected the antioxidative enzymes (SOD, GSH-Px, CAT, and T-AOC) in ALI mice (*p* < 0.05). These results indicating *L. paracasei* CCFM1223 relieve the development of ALI by reducing inflammation and elevating antioxidative enzymes.

**Figure 3:**
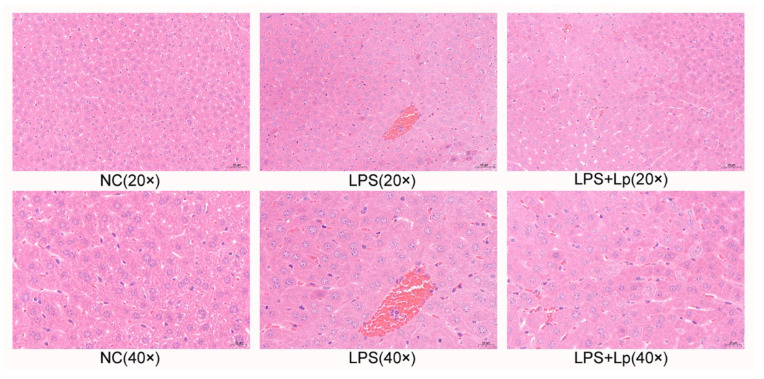
Representative H&E staining of liver sections (scale bar: 50 µm).

As shown in Figure 3, no obvious histologic lesions of liver tissues were presented in the NC group. The clearly inflammatory infiltration, clearly visible nucleus, cytoplasmic or vacuolar swelling, and balloon degeneration of liver tissues were observed in the LPS group. However, these appearances were obviously ameliorated in ALI mice with *L. paracasei* CCFM1223 pretreatment. These data exhibited that *L. paracasei* CCFM1223 pretreatment mitigated LPS-induced ALI.

### 3.4. L. paracasei CCFM1223 Pretreatment Shifted the SCFAs in ALI Mice

**Figure 4:**
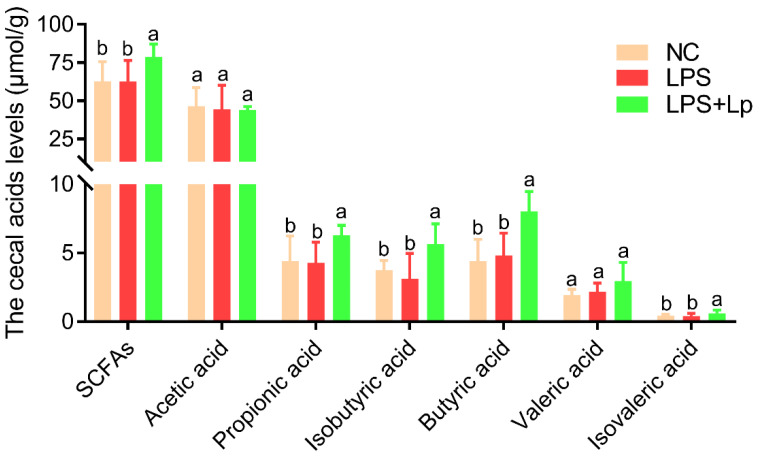
Effect of *L. paracasei* CCFM1223 pretreatment on the cecal SCFAs levels in LPS-treated mice (n = 8). Values with different letters are significantly different (*p* < 0.05).

The intestinal microbial metabolites played essential roles in improving the host immune system, especially SCFAs. Therefore, the regulation influence of *L. paracasei* CCFM1223 on the cecal SCFAs levels was further analyzed in this study (Figure 4). No clear alters in the cecal SCFAs levels were presented between the NC and LPS groups (*p* > 0.05). *L. paracasei* CCFM1223 intervention significantly elevated the cecal SCFAs levels, especially propionic, isobutyric, butyric, valeric, and isovaleric acids (*p* < 0.05). Nevertheless, the cecal acetic acid levels in ALI mice were not restored by *L. paracasei* CCFM1223 intervention. Thus, *L. paracasei* CCFM1223 relieves the development of ALI, possibly by elevating the concentrations of cecal SCFAs.

### 3.5. L. paracasei CCFM1223 Pretreatment Reshaped the Intestinal Microbiota in ALI Mice

**Figure 5:**
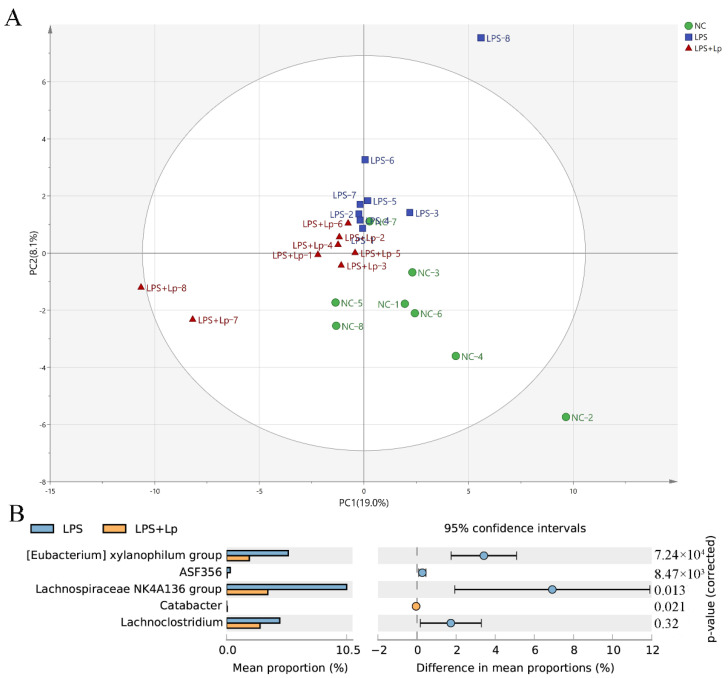
Effect of *L. paracasei* CCFM1223 pretreatment on the intestinal microbiota structure in LPS-treated mice (n = 8). Primary component analysis (PC1 × PC2) based on weighted UniFrac (**A**). Relative abundance of differential microorganisms between LPS and LPS+Lp groups (**B**).

The overall structure of the intestinal microbiota of mice in the NC, LPS, and LPS+Lp groups was analyzed. The first two main components of PCA accounted for 19.0% and 8.1% of the total data change, respectively (Figure 5A). The PCA results exhibited an obvious distinction between the NC and LPS groups in the composition of the intestinal microbiota of mice. However, the structure of intestinal microbiota in the LPS+Lp group moved in the direction of the NC group and went away from the LPS group, indicating *L. paracasei* CCFM1223 could shift the intestinal microbiota of the LPS mice. The clear distinction among the three groups was observed by the clustering result (Figure S4).

Figure S5 exhibits the specific composition of the intestinal microbiota community at the genus level. The relative abundance of [*Eubacterium*] *ventriosum group*, *Ruminiclostridium 5*, *Enterorhabdus*, *Escherichia*-*Shigella* in the LPS group were remarkably decreased compared with the NC group, while the relative abundance of *Lachnoclostridium*, *Prevotellaceae NK3B31 group* were remarkably increased (*p* < 0.05). However, *L*. *paracasei* CCFM1223 intervention remarkably reduced the relative abundance of [*Eubacterium*] *xylanophilum group*, *ASF356*, *Lachnospiraceae NK4A136 group*, and *Lachnoclostridium* and remarkably increased the relative abundance of *Catabacter* compared with the LPS group (*p* < 0.05) (Figure 5B). It was suggested that *L. paracasei* CCFM1223 remarkably improves the structure of intestinal microbiota.

### 3.6. Correlation of Key Intestinal Microbiota with ALI Indicators

**Figure 6:**
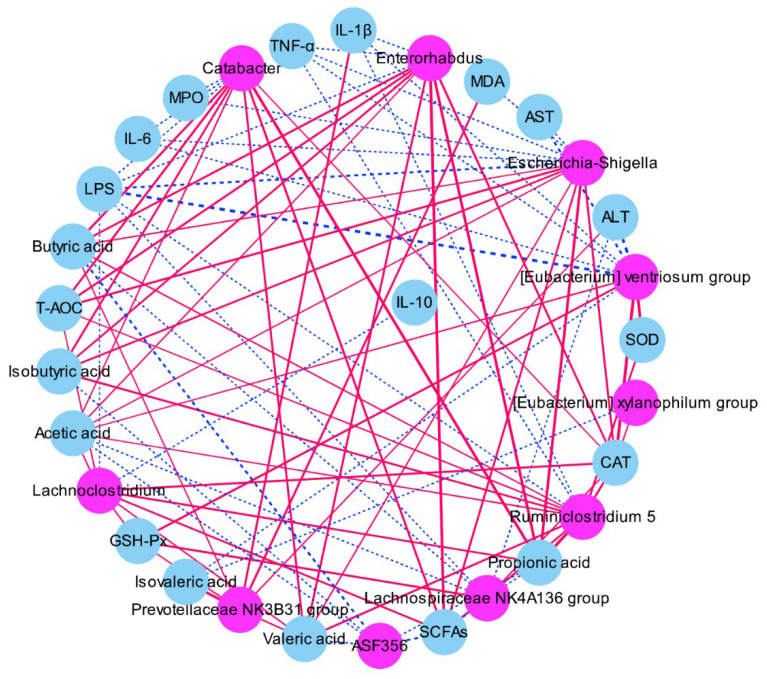
Correlation network of spearman’s correlations analysis between the key intestinal bacterial phylotypes and parameters of ALI. Red shows positive association, and blue shows negative association.

In order to explore the regulatory direction of these intestinal microbiotas with remarkable alters on the anti-inflammation function of mice, the association between the key bacterial genera and the important indexes-related ALI was analyzed based on the Spearman correlation analysis (Figure 6 and Figure S6). *Catabacter*, *Enterorhabdus*, *Escherichia*-*Shigella*, *Lachnoclostridium*, *Ruminiclostridium* 5, and *Prevotellaceae NK3B31 group* mainly exhibited a positive correlation with the cecal SCFAs levels (propionic acid, butyric acid, acetic acid, lsobutyric acid, and valeric acid), antioxidative enzymes activities (T-AOC, GSH-Px, SOD, and CAT), and a negative correlation with the hepatic inflammation cytokines (IL-1β, IL-6, TNF-α, and LPS), the serum biochemical index (ALT and AST). On the contrary, [*Eubacterium*] *xylanophilum group*, *ASF356*, *Lachnospiraceae NK4A136 group* mainly exhibited a negative correlation with the cecal SCFAs levels (propionic acid, butyric acid, acetic acid, isobutyric acid, and valeric acid) and inflammation cytokines (IL-1β, IL-6, TNF-α, and LPS), whereas it mainly exhibited a positive correlation with the activities of antioxidative enzymes (GSH-Px, SOD, and CAT).

### 3.7. L. paracasei CCFM1223 Alters the Expression of Genes in ALI Mice

**Figure 7:**
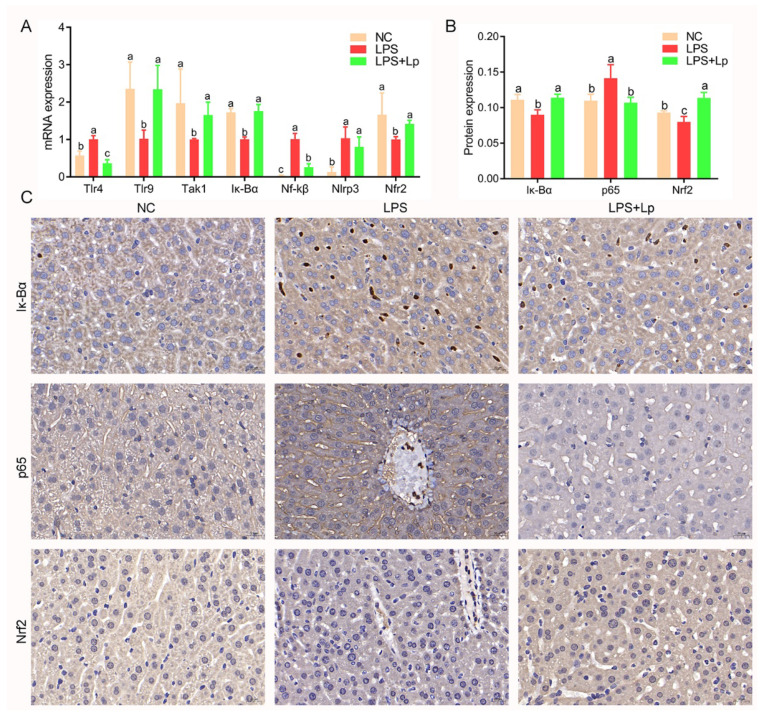
Effect of *L. paracasei* CCFM1223 on the expression of gene-related inflammation and oxidative stress in LPS-treated mice. mRNA expression for Tlr4, Tlr9, Tak1, Iκ-Bα, Nf-kβ, Nlrp3, and Nrf2 in liver (**A**). Immunohistochemical staining for Nf-kβ, p65, and Nrf2 in liver (**B**). The mean density of Nf-kβ, p65, and Nrf2 in liver (**C**). Values with different letters are significantly different (*p* < 0.05).

To further explore the hepatoprotective efficacy of *L. paracasei* CCFM1223 on liver signaling in ALI mice, the expression of genes-related ALI was determined using RT-qPCR. As described in Figure 7A, intraperitoneal injection of LPS remarkably up-regulated the transcriptions of hepatic Tlr4 and Nf-kβ, whereas remarkably down-regulated the transcriptions of hepatic Tlr9, Tak1, Iκ-Bα, and Nrf2, compared with mice without LPS treatment (*p* < 0.05). In contrast, these changes induced by LPS were remarkably reversed after *L. paracasei* CCFM1223 pretreatment (*p* < 0.05), whereas the transcription of hepatic Nlrp3 was slightly reduced in ALI mice (*p* < 0.05). In addition, the results of the immunohistochemical analysis also displayed that *L. paracasei* CCFM1223 pretreatment significantly up-regulated the Iκ-Bα and Nrf2 expressions but significantly down-regulated the p65 expression in the liver (*p* < 0.05) (Figure 7B,C).

## 4. Discussion

Some evidence has shown that the occurrence of ALI is strongly associated with inflammation and oxidative stress [15,16]. Nevertheless, it is unclear how *Lactobacillus* prevent the occurrence of ALI and relieve the symptom of ALI. Further study is necessary on how *Lactobacillus* regulates the inflammation and oxidative stress-related ALI, particularly the gut–liver axis. The hepatoprotective mechanism of *L. paracasei* CCFM1223 in ALI was revealed in this study by endotoxin replicating the symptom of ALI. The results of this study displayed that *L. paracasei* CCFM1223 attenuates LPS-induced abnormal inflammation and oxidative stress in mice.

It is reported that long-term *Lactobacillus* consumption is beneficial for high-fat diet-induced abnormal serum lipid levels, including TC, TG, LDL-C, and HDL-C [17]. As everyone knows, TC and LDL-C serve as the vital risk factors for cardiovascular disease, and a 1% increase in serum TC concentration elevates the risk of cardiovascular disease (approximately 3%) [18]. At present, some reports exhibited that long-time *Lactobacillus* intake effectively ameliorative body weight and glycolipid levels in hyperlipidemic mice [6,19]. Our results also found that *L. paracasei* CCFM1223 pretreatment remarkably decreased the serum TC and LDL-C levels in ALI mice, indicating that *L. paracasei* CCFM1223 has the capacity to ameliorate the host lipid metabolism. In addition, the serum ALT and AST activities are wide-range applied for assessing liver function because they are transferred into the blood circulation when the liver injury occurs. According to the investigation by World Health Organization (WHO), ALT serves as the most sensitive parameter of liver structure injury, and the serum ALT mainly stems from the damage to the cell membrane [20]. A previous study displayed that the serum ALT and AST activities were remarkably increased in LPS-treated liver-injury mice, which is in agreement with this study [2]. Nevertheless, *L. paracasei* CCFM1223 pretreatment remarkably suppressed the serum AST and ALT activities compared with the LPS group, suggesting that *L. paracasei* CCFM1223 had a protective effect against ALI. In addition, it is widely accepted that excessive oxidative stress is one of the most important factors in the pathogenesis of liver injury. A previous study suggested that intraperitoneal injection of LPS results in abnormal levels of oxidative stress in ALI mice [21]. MDA serves as the major end product of lipid peroxidation, disseminates and aggravates oxidative liver damage, and is beneficial for exploring the extent of damage to the biological membrane [22]. Thus, decreasing hepatic MDA levels is an effective approach to relieving oxidative stress. Here, we observed that *L. paracasei* CCFM1223 pretreatment against LPS treatment induced the elevation of hepatic MDA level and the reduction of antioxidative enzyme activities (T-AOC, SOD, GSH-Px, and CAT). The action of SOD is the inhibition of reactive oxygen species (ROS) and the boost of hydrogen peroxide formation in vivo, which was further decomposed to non-toxic substances (oxygen and water) under the higher activity of GSH-Px [23]. LPS intraperitoneal injection accelerates the production of ROS from inflammatory cells, which further aggravates ALI and leads to chronic inflammation. Whereas CAT serves as one of the primary ROS scavengers, and thus activation of CAT accelerates the elimination of ROS [24]. Our results display that the blockade of LPS-induced oxidative stress is associated with the amelioration of ALI.

In recent years, “gut–liver” axis has received wide-range attention in the world. Gut–liver axis is composed of intestinal microbiota, intestinal barrier, and liver function. Our study showed that *L. paracasei* CCFM1223 pretreatment obviously altered the intestinal flora composition, including [*Eubacterium*] *ventriosum group*, *Ruminiclostridium* 5, *Enterorhabdus*, *Escherichia*-*Shigella*, *Lachnoclostridium*, *Prevotellaceae NK3B31 group*, [*Eubacterium*] *xylanophilum group*, *ASF356*, *Lachnospiraceae NK4A136 group*, *Lachnoclostridium*, and *Catabacter*. [*Eubacterium*] *Ventriosum group* as genus level of Eubacteriaceae family has been considered as butyrate-producing bacteria, and negatively associated with the inflammation cytokines (IL-6 and TNF-α), which is in agreement with this study [25]. Studies have reported that *Ruminiclostridium* [5], belonging to Firmicutes, which can produce SCFAs by fermented carbohydrates, demonstrate an ability to supply energy for intestinal cells, and maintain the gut barrier integrity [26]. *Enterorhabdus* is a member of the family Coriobacteriaceae that occurs widely in the feces and intestines of mammals [27]. *Catabacter* is suggested to exert protective effects against liver function injury by producing cecal butyric acids [28]. However, *Lachnoclostridium* has been found in stools of Hashimoto’s thyroiditis patients and is positively associated with chronic inflammation [29]. *Prevotellaceae NK3B31 group* belonged to Prevotellaceae, and it has been proven that there is a significant positive correlation with the TNF-α levels, which is similar to our data [30]. In addition, long-time consumption of a high-energy diet increased the abundance of the *Lachnospiraceae NK4A136 group*, which has been reported to increase obesity and colitis in animals [31]. These results indicated that *L. paracasei* CCFM1223-induced selective increase and reduction of intestinal microorganisms may be contributed to the hepatoprotective effects of *L. paracasei* CCFM1223.

SCFAs are a group of fatty acids containing less than six carbons that mainly stem from undigested polysaccharides, and their concentration is opposite associated with the pathogenesis of many diseases, such as autoimmune diseases, metabolic diseases, and neurological diseases. Our study exhibited that *L. paracasei* CCFM1223 treatment remarkably elevated the cecal SCFAs levels, including propionic, isobutyric, butyric, valeric, and isovaleric acids. Among them, butyric acid promotes the IL-22 production in the intestines by CD^4+^ T cell and ILCs via combining the G-protein receptor 41(GPR41) and activating aryl hydrocarbon receptor (AhR) and hypoxia-inducible factor 1α (HIF-1α) [32]. High concentrations of IL-22 promote the haptoglobin LPS binding protein and serum amyloid A production [33]. However, a previous report has shown that gut-derived IL-22 maintained the integrity of the intestinal barrier and elevated the host immune system [13]. In the present study, the colonic, serum, and hepatic IL-22 levels were remarkably elevated in the LPS+Lp group compared with the NC and LPS group, indicating that *L. paracasei* CCFM1223 intervention accelerates the production of colonic IL-22 by elevating the concentration of butyric acid and then regulate the systemic level of IL-22. Hepatic IL-22 accelerates the recruitment of regulatory cDCs to the liver and further promotes the production of IL-22 and TGF-β by combining hepatic Tlr9 [13]. In addition, intraperitoneal injection of LPS elevated the hepatic LPS concentration and then activated the expression of Tlr4, which is a type I transmembrane protein. Overexpression of hepatic Tlr4 help to elevate the expression of Tlr9, which is in agreement with our results [34]. TGF-β promotes the recruitment, differentiation, and proliferation of immune cells and further suppresses the occurrence and development of inflammation in the liver [35]. Higher levels of hepatic IL-22 and TGF-β suppressed the production of TNF-α and the expression of Nf-kβ by up-regulating the expression Tak1 in the liver. TNF-α serves as a vital regulator of immune regulation and inflammation that direct cytotoxic effect and induce hepatocyte necrosis [36]. The secretion of TNF-α is monitored by the levels of IL-1β that one of the most cell cytokines and plays an important role in the so-called cytokine storm. Activation of the Nf-kβ pathway promotes the development of ALI by elevating the secretion of inflammatory cytokines, including IL-1β and IL-6 [37]. IL-6 is traditionally considered a regulator of acute-phase responses, and its levels are strongly associated with cardiovascular disease, type 2 diabetes, and liver functional decline [38]. In the present study, the hepatic TNF-α, IL-1β, and IL-6 levels were remarkably decreased in ALI mice after the pretreatment of *L. paracasei* CCFM1223. In addition, Nf-kβ serves as a pivotal controller in the Nlrp3 inflammasome in the liver [39]. Activation of Nlrp3 promotes the production, maturation, and recruitment of IL-1β. Our results exhibited that *L. paracasei* CCFM1223 pretreatment down-regulated the transcription of hepatic Tlr4, Nf-kβ, and Nlrp3 and up-regulated the transcription of hepatic Tlr9 and Tak1 in ALI mice. According to these results, we can speculate that *L. paracasei* CCFM1223 attenuates the inflammation in ALI mice may by stimulating the intestinal IL-22 production and further regulating the expression of inflammation-related genes.

## 5. Conclusions

The potential influence of *L. paracasei* CCFM1223 on LPS-induced ALI and their possible mechanisms were firstly investigated. The possible protective mechanisms of *L. paracasei* CCFM1223 against ALI were elucidated using 16S rRNA amplicon high throughput sequencing and RT-qPCR. These results preliminarily indicate that *L. paracasei* CCFM1223 remarkably suppressed the hepatic pro-inflammation cytokines production and remarkably elevated the antioxidative enzymes in ALI mice by shifting the intestinal microbiota composition, stimulating the colonic IL-22 production, and inhibiting the expressions of inflammation-related genes. In conclusion, this study implied that *L. paracasei* CCFM1223 has a beneficial influence on treating endotoxemia-induced ALI and is expected to become a new food supplement. In addition, further study is needed to clarify the vital role of IL-22 in the hepatoprotective effect of *L. paracasei* CCFM1223 in ALI patients using germ-free mice and gene knockout technology, which will supply more credible information to the clinical application.

## Figures and Tables

**Table 1:** The effects of *L. paracasei* CCFM1223 on the cell cytokines in ALI mice (n = 8).

	NC	LPS	LPS+Lp
TNF-α (pg/mg prot.)	33.82 ± 13.52 ^c^	59.71 ± 20.71 ^a^	41.42 ± 6.38 ^b^
IL-1β (pg/mg prot.)	18.12 ± 7.40 ^c^	50.23 ± 12.02 ^a^	28.36 ± 5.38 ^b^
IL-6 (pg/mg prot.)	7.85 ± 2.45 ^a^	10.54 ± 3.64 ^a^	8.19 ± 1.04 ^a^
IL-17 (pg/mg prot.)	72.16 ± 18.54 ^b^	122.76 ± 41.84 ^a^	109.33 ± 17.04 ^a^
IL-10 (pg/mg prot.)	295.09 ± 72.99 ^a^	196.71 ± 83.54 ^b^	328.35 ± 43.48 ^a^
TGF-β (pg/mg prot.)	10.48 ± 0.16 ^a^	4.76 ± 2.05 ^b^	13.42 ± 3.52 ^a^
LPS (EU/mg prot.)	1.57 ± 0.46 ^b^	3.13 ± 0.49 ^a^	2.15 ± 0.54 ^b^
MDA (nmol/mg prot.)	1.03 ± 0.33 ^b^	1.82 ± 0.65 ^a^	0.79 ± 0.30 ^b^
SOD (U/mg prot.)	3.14 ± 1.2 ^a^	0.90 ± 0.84 ^b^	2.61 ± 2.13 ^ab^
GSH-Px (U/mg prot.)	23.36 ± 8.94 ^ab^	16.71 ± 5.96 ^b^	25.87 ± 7.18 ^a^
CAT (U/mg prot.)	2.74 ± 0.92 ^a^	1.10 ± 0.13 ^b^	2.72 ± 0.26 ^a^
T-AOC (U/mg prot.)	23.36 ± 8.93 ^a^	16.71 ± 5.96 ^c^	25.87 ± 7.18 ^b^

Values with different letters are significantly different (*p* < 0.05).

## Data Availability

The data shown in the present research are available on request from the corresponding author.

## Supplementary Materials

The following supporting information can be downloaded at: https://www.mdpi.com/article/10.3390/microorganisms10071321/s1, Figure S1. Animal model experimental design; Figure S2: Effect of *L. paracasei* CCFM1223 on body weight in LPS-treated mice (n = 8); Figure S3: Effect of *L. paracasei* CCFM1223 on the serum TC, TG, HDL-C, and LDL-C levels in LPS-treated mice (n = 8); Figure S4: Hierarchical cluster analysis based on the genus level; Figure S5: Extended error bar plot identifying the intestinal microbiota of significant differences between NC and LPS groups; Figure S6: Heatmap of Spearman’s correlation analysis between the key intestinal bacterial phylotypes and parameters of ALI; Table S1: Primer sequences for quantitative real-time PCR of hepatic genes.
