# Supplementary material for: Lactobacillus paracasei CCFM1223 Protects against Lipopolysaccharide-Induced Acute Liver Injury in Mice by Regulating the “Gut–Liver” Axis
Source: Microorganisms. 2022 Jun 30;10(7):1321. doi: 10.3390/microorganisms10071321 (PMC9319883; doi:10.3390/microorganisms10071321)
Supplement: Supplementary file 1 [file microorganisms-10-01321-s001.zip › microorganisms-1773741-supplementary.pdf]

Supplementary Materials

Supplementary Material 1:

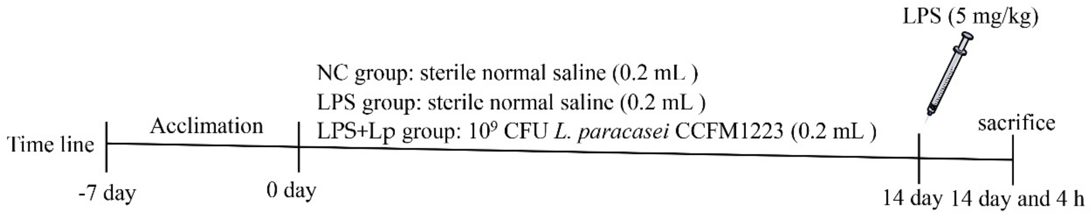

Figure S1. Animal model experimental design.

Supplementary Material 2

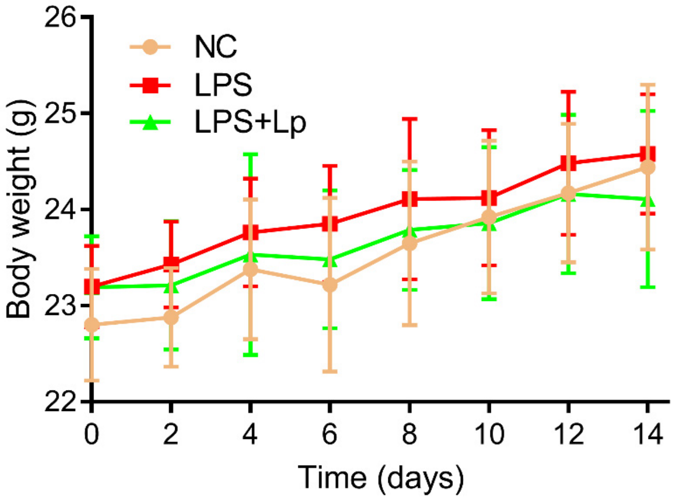

Figure S2 Effect of *L. paracasei* CCFM1223 on body weight in LPS-treated mice (n = 8）.

Supplementary Material 3:

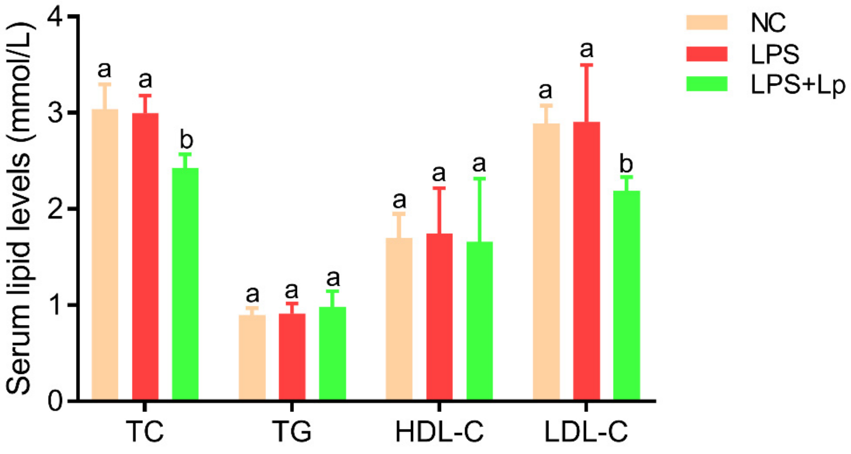

Figure S3 Effect of *L. paracasei* CCFM1223 on the serum TC, TG, HDL-C, and LDL-C levels in LPS-treated mice (n = 8). Values with different letters are significantly different ($p < 0.05$).

Supplementary Material 4:

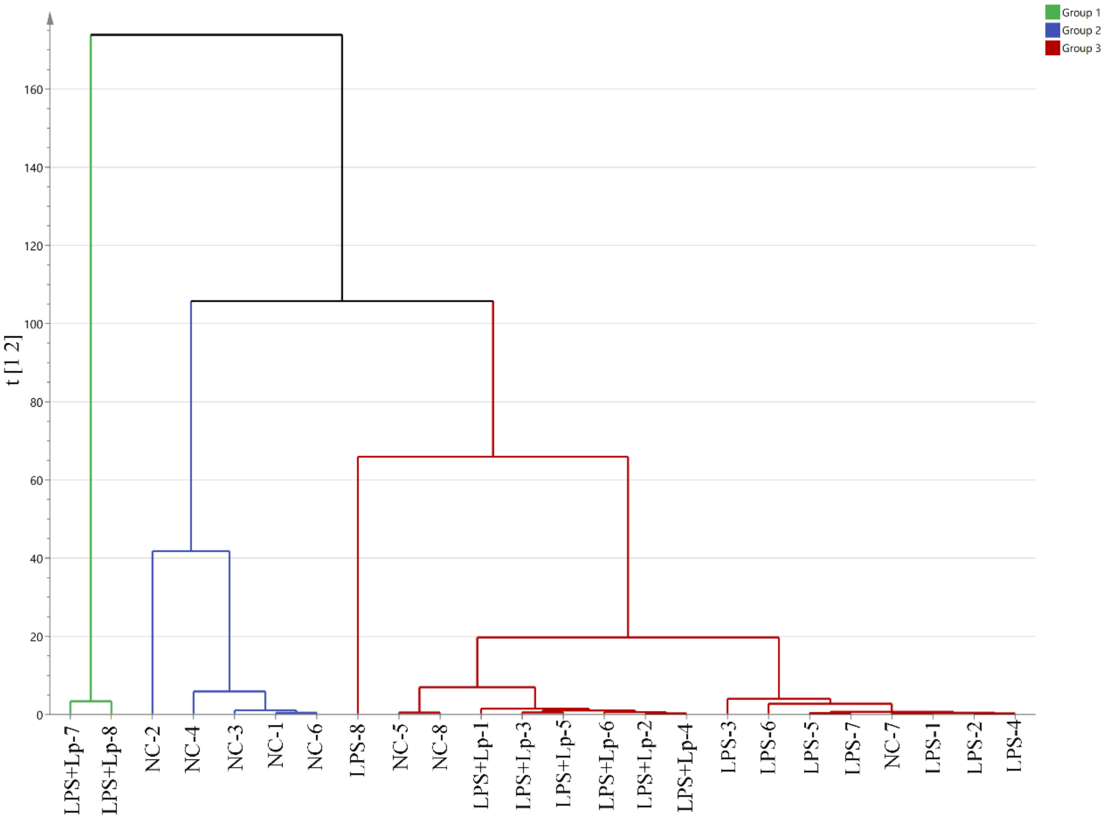

Figure S4 Hierarchical cluster analysis based on the genus level.

Supplementary Material 5:

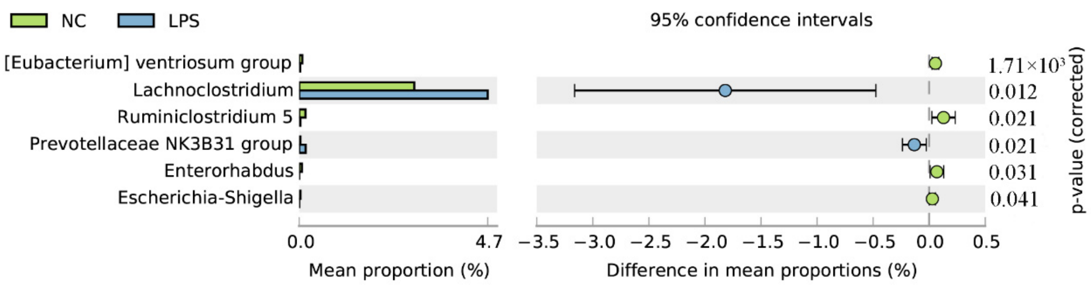

Figure S5 Extended error bar plot identifying the intestinal microbiota of significant differences between NC and LPS groups.

Supplementary Material 6:

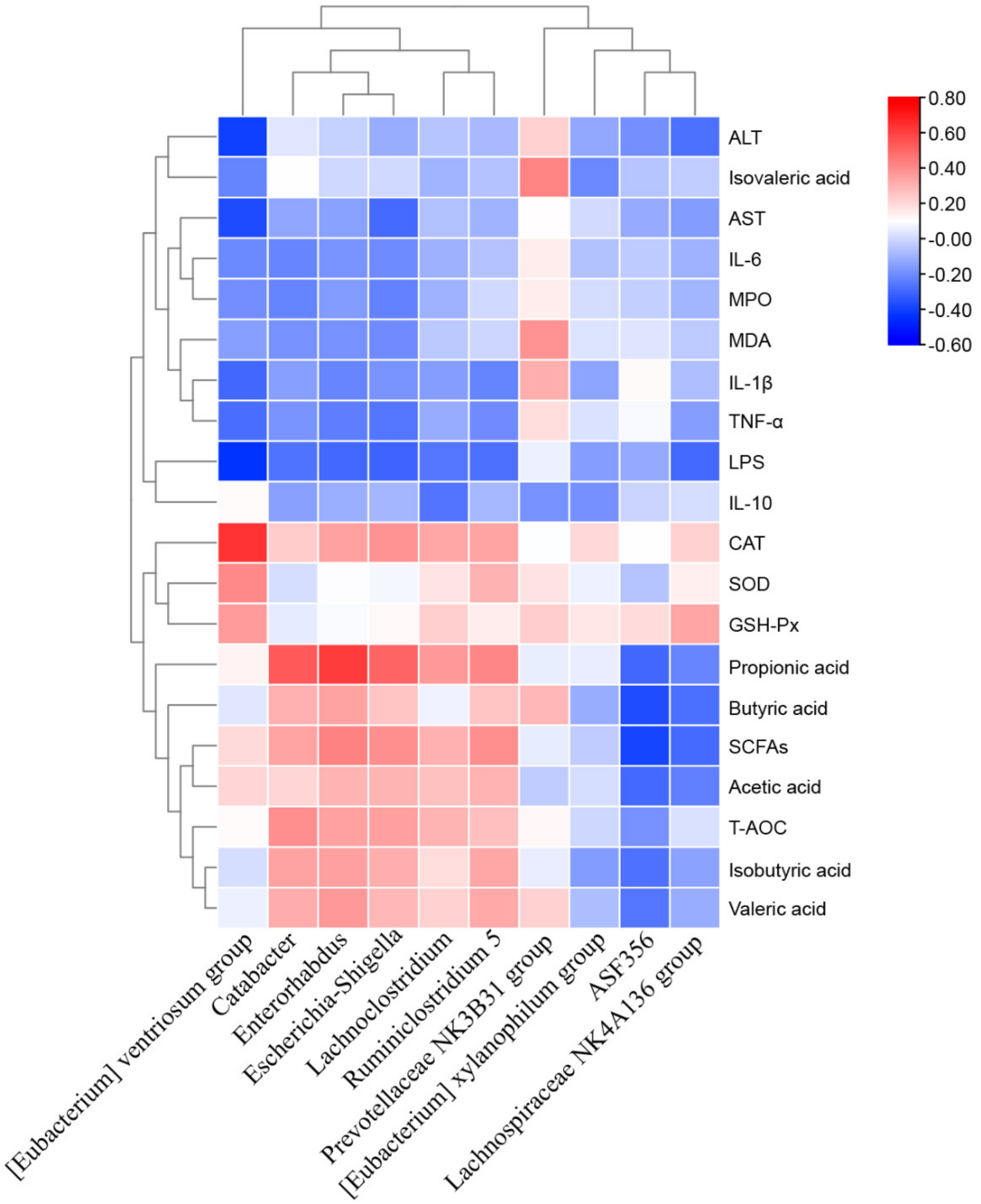

Figure S6 Heatmap of Spearman's correlation analysis between the key intestinal bacterial phylotypes and parameters of ALI.

Supplementary Material 7:

Table S1 Primer sequences for quantitative real-time PCR of hepatic genes.

Primers	Forward Sequences (5′→3′)	Reverse Sequences (5′→3′)
Tlr4	GGCAGCAGGTGGAATTGTAT	AGGCCCCAGAGTTTTGTTCT
Tlr9	CTCCAACCGTATCCACCACC	GAGAAGTGCAGGGGGCTAAG
Nfr2	CCTCCGCTGCCATCAGTCAGT	TCGGCTGGGACTCGTGTTCA
Tak1	TCAGCACGTTGATCGTTGGT	TCCATCTGGGCTGGTTAGGA
Iκ-Bα	ACCAACCAGCCAGAAATCG	TCACAGGCAAGGTGTAGAGGG
Nf-kβ	CGCCCCTTATCGACCACC	CCTTCTCCCAAGAGTCGTCCA
Nlrp3	CCCTTTATTTGTACCCAAGGCT	CGGGCGGGTAATCTTCCAAA
β-actin	GCACCACACCTTCTACAATG	TGCTTGCTGATCCACATCTG
